# Correction: Can network attention effectively stimulate corporate ESG practices?—Evidence from China

**DOI:** 10.1371/journal.pone.0318880

**Published:** 2025-02-03

**Authors:** The PLOS ONE Staff

The second author, Shuang Cao, is incorrectly noted as the corresponding author. The correct corresponding author is En Xie. En Xie email address is: xenzi2022@163.com.

The publisher apologizes for the error.
