# Supplementary material for: Can network attention effectively stimulate corporate ESG practices?—Evidence from China
Source: PLoS One. 2023 Dec 14;18(12):e0290993. doi: 10.1371/journal.pone.0290993 (PMC10721026; doi:10.1371/journal.pone.0290993)
Supplement: Attachment — Submitted filename: PONE-D-23-10880 review.pdf [file pone.0290993.s002.pdf]

PLOS ONE

Can network attention effectively stimulate corporate ESG practices? — Evidence from China

--Manuscript Draft--

**Manuscript Number:**	PONE-D-23-10880
**Article Type:**	Research Article
**Full Title:**	Can network attention effectively stimulate corporate ESG practices? — Evidence from China
**Short Title:**	Can network attention effectively stimulate corporate ESG practices?
**Corresponding Author:**	en xie<br>Hainan University<br>haikou, hainan CHINA
**Keywords:**	Network attention; ESG; Legitimacy; Inspiration; Financing constraints
**Abstract:**	ESG has emerged as a widespread concern for all societal segments. We aim to explore the influence of network attention on corporate ESG practices from an investor perspective. We find that rising network attention significantly increases corporate ESG practices. Specifically, network attention plays the role of external monitoring, image promotion incentives, and mitigation of financing constraints to make companies willing to challenge ESG practices. Additionally, the promoting effect of network concerns on firms' ESG practices was more significant in higher marketization processes, severely competitive industries, and non-state enterprises. In the Internet era, companies must pay attention to the flow effect caused by network attention, meet stakeholder demands, and pursue long-term sustainable development.
**Order of Authors:**	en xie
**Additional Information:**	
**Question**	**Response**
**Financial Disclosure**<br><br>Enter a financial disclosure statement that describes the sources of funding for the work included in this submission. Review the submission guidelines for detailed requirements. View published research articles from *PLOS ONE* for specific examples.<br><br>This statement is required for submission and **will appear in the published article** if the submission is accepted. Please make sure it is accurate.	The author received no specific funding for this work.

Powered by Editorial Manager® and ProduXion Manager® from Aries Systems Corporation

**Unfunded studies**<br>Enter: *The author(s) received no specific funding for this work.*<br><br>**Funded studies**<br>Enter a statement with the following details:<br>• Initials of the authors who received each award<br>• Grant numbers awarded to each author<br>• The full name of each funder<br>• URL of each funder website<br>• Did the sponsors or funders play any role in the study design, data collection and analysis, decision to publish, or preparation of the manuscript?<br>• **NO** - Include this sentence at the end of your statement: *The funders had no role in study design, data collection and analysis, decision to publish, or preparation of the manuscript.*<br>• **YES** - Specify the role(s) played.<br><br>* typeset	
**Competing Interests**<br><br>Use the instructions below to enter a competing interest statement for this submission. On behalf of all authors, disclose any competing interests that could be perceived to bias this work—acknowledging all financial support and any other relevant financial or non-financial competing interests.<br><br>This statement is required for submission and **will appear in the published article** if the submission is accepted. Please make sure it is accurate and that any funding sources listed in your Funding Information later in the submission form are also declared in your Financial Disclosure statement.<br><br>View published research articles from *PLOS ONE* for specific examples.	The authors have declared that no competing interests exist.

**NO authors have competing interests**<br><br>Enter: *The authors have declared that no competing interests exist.*<br><br>**Authors with competing interests**<br><br>Enter competing interest details beginning with this statement:<br><br>*I have read the journal's policy and the authors of this manuscript have the following competing interests: [insert competing interests here]*<br><br>* typeset	
**Ethics Statement**<br><br>Enter an ethics statement for this submission. This statement is required if the study involved:<br><br>• Human participants<br>• Human specimens or tissue<br>• Vertebrate animals or cephalopods<br>• Vertebrate embryos or tissues<br>• Field research<br><br>Write "N/A" if the submission does not require an ethics statement.<br><br>General guidance is provided below. Consult the submission guidelines for detailed instructions. **Make sure that all information entered here is included in the Methods section of the manuscript.**	N/A

Powered by Editorial Manager® and ProduXion Manager® from Aries Systems Corporation

**Format for specific study types**<br><br>**Human Subject Research (involving human participants and/or tissue)**<br>• Give the name of the institutional review board or ethics committee that approved the study<br>• Include the approval number and/or a statement indicating approval of this research<br>• Indicate the form of consent obtained (written/oral) or the reason that consent was not obtained (e.g. the data were analyzed anonymously)<br><br>**Animal Research (involving vertebrate animals, embryos or tissues)**<br>• Provide the name of the Institutional Animal Care and Use Committee (IACUC) or other relevant ethics board that reviewed the study protocol, and indicate whether they approved this research or granted a formal waiver of ethical approval<br>• Include an approval number if one was obtained<br>• If the study involved *non-human primates*, add *additional details* about animal welfare and steps taken to ameliorate suffering<br>• If anesthesia, euthanasia, or any kind of animal sacrifice is part of the study, include briefly which substances and/or methods were applied<br><br>**Field Research**<br><br>Include the following details if this study involves the collection of plant, animal, or other materials from a natural setting:<br>• Field permit number<br>• Name of the institution or relevant body that granted permission	
**Data Availability**<br><br>Authors are required to make all data underlying the findings described fully available, without restriction, and from the time of publication. PLOS allows rare exceptions to address legal and ethical concerns. See the PLOS Data Policy and FAQ for detailed information.	Yes - all data are fully available without restriction

Powered by Editorial Manager® and ProduXion Manager® from Aries Systems Corporation

A Data Availability Statement describing where the data can be found is required at submission. Your answers to this question constitute the Data Availability Statement and **will be published in the article**, if accepted.<br><br>**Important:** Stating 'data available on request from the author' is not sufficient. If your data are only available upon request, select 'No' for the first question and explain your exceptional situation in the text box.<br><br>Do the authors confirm that all data underlying the findings described in their manuscript are fully available without restriction?	
**Describe where the data may be found in full sentences. If you are copying our sample text, replace any instances of XXX with the appropriate details.**<br><br>• If the data are **held or will be held in a public repository**, include URLs, accession numbers or DOIs. If this information will only be available after acceptance, indicate this by ticking the box below. For example: *All XXX files are available from the XXX database (accession number(s) XXX, XXX.*).<br>• If the data are all contained **within the manuscript and/or Supporting Information files**, enter the following: *All relevant data are within the manuscript and its Supporting Information files.*<br>• If neither of these applies but you are able to provide **details of access elsewhere**, with or without limitations, please do so. For example:<br><br>*Data cannot be shared publicly because of [XXX]. Data are available from the XXX Institutional Data Access / Ethics Committee (contact via XXX) for researchers who meet the criteria for access to confidential data.*<br><br>*The data underlying the results presented in the study are available from (include the name of the third party*	The network attention are available from the CNRDS database,and financial data of the company from the China Stock Market and Accounting Research (CSMAR).

and contact information or URL).<br>• This text is appropriate if the data are owned by a third party and authors do not have permission to share the data.<br><br>* typeset	
Additional data availability information:	Tick here if the URLs/accession numbers/DOIs will be available only after acceptance of the manuscript for publication so that we can ensure their inclusion before publication.

anonymous Can network attention effectively stimulate corporate ESG practices
Click here to access/download;Manuscript;anonymous .docx

En Xie

School of Management, Hainan University, Haikou 570228, China.Email: xenzi2022@163.com.

Can network attention effectively stimulate corporate ESG practices? — Evidence from China

Abstract: ESG has emerged as a widespread concern for all societal segments. We aim to explore the influence of network attention on corporate ESG practices from an investor perspective. We find that rising network attention significantly increases corporate ESG practices. Specifically, network attention plays the role of external monitoring, image promotion incentives, and mitigation of financing constraints to make companies willing to challenge ESG practices. Additionally, the promoting effect of network concerns on firms' ESG practices was more significant in higher marketization processes, severely competitive industries, and non-state enterprises. In the Internet era, companies must pay attention to the flow effect caused by network attention, meet stakeholder demands, and pursue long-term sustainable development.

Keywords: Network attention; ESG; Legitimacy; Inspiration; Financing constraints

1. Introduction

China's economy has shifted from a phase of high growth to high-quality development. Climate change, depletion of natural resources, adverse working conditions, and corporate scandals have heightened society's expectations of corporate environmental, social and ethical responsibility (Barber and Odean 2008). The United Nations Global Compact (UNGC) first introduced the concept of ESG in June 2004, advocating that companies should consider the unified development of the environment, society and governance while focusing on business, going beyond the classic financial performance indicators. In March 2021, China proposed the "double carbon" target of achieving "peak carbon" by 2030 and "carbon neutrality" by 2060. Simultaneously, the executive

meeting of the State Council also proposed to strengthen the quality of listed companies further, perfect the governance rules of listed companies and enhance the transparency of information as well as the quality of disclosure. As such, it is evident that the Philosophy of ESG has coincided with the above macro requirements. ESG practices have emerged as a core corporate decision, garnering widespread attention from consumers, investors, and other stakeholders. However, regarding China's investment structure, there are relatively fewer institutional investors, and retail investors predominate the majority. Retail investors, who are at an information disadvantage, actively search the internet to find out information about companies in order to make accurate investment decisions. With the inevitably rapid development of China’s internet technology, the size of China’s internet users reached 1.032 billion as of December 2021, with an Internet penetration rate of 73%[1]. According to the latest global statistics from the U.S. national statistics agency, Baidu’s search engine commanded a 74.63% market share by the end of February 2019[2]. Therefore, the search data can reflect the investors’ concerns about capital market transactions and demand, which can function as the most direct and objective evidence of retail investors’ online attention. As such, the internet has inevitably entered the era where “attaining traffic implies being the king”. Once a company enters the public eye and becomes the object of attention of investors or even the general public, then the network attention will gradually become a traffic resource which will significantly affect the investment decision of the company (Deng, Feng, and Li 2020). As such, we seek to uncover the influences that drive companies’ ESG strategies that are essential for enhancing corporate sustainability. In fact, the informal system has long been a neglected and

---

1 The 46th "Statistical Report on the Development Status of the Internet in China" released by CNNIC

2 https://gs.statcounter.com/

critical variable in driving ESG management and practice. In China's imperfect legal market, network attention, as a kind of informal regulation, represents a powerful complement to the formal system and serves an essential role in enhancing the level of corporate governance and protecting stakeholders. Confronted with the mobile internet traffic effect as an emerging shock, scholars have thoroughly examined the implications of investor concerns on corporate green innovation. However, there is still relatively limited research on the impact of network attention on firms' decision-making behaviour (Li et al. 2022). As such, we research the impact of network attention on corporate ESG practices, supplementing to this part of the literature.

In view of this, we empirically examine the impact of network attention on corporate ESG practices. We discovered that the network attention had positively contributed to corporate ESG practices regardless of whether ESG has been viewed as a whole or broken down into its three sub-dimensions of E、S and G. We believe that the network attention on external governance effects and internal incentives has strengthened the enterprise's willingness to practice ESG. Simultaneously, the network attention can effectively alleviate financing constraints and improve the enterprise's ESG practice ability. Furthermore, expand and analyze whether the relationship between network attention and ESG is different in different situations at the national level, industry level as well as enterprise level. According to the result, the positive correlation between network attention and ESG practice is more significant in highly market-oriented, highly competitive industries, as well as non-state-owned enterprises.

Our predominant potential contributions are in the following three aspects. Firstly, the existing ESG research has primarily focused on its impact on companies in developed markets with China being a relative latecomer in the field of ESG research. Also, we have utilized the web search index which is calculated based on the Baidu

index as a proxy variable for internet attention, enriching the emerging market on ESG validation. Also, it has more often been oriented towards a single dimension. While regarding ESG as a whole, we divide it into three subcategories (environment, society and governance), to provide more detailed evidence for the impact of attention towards network on specific ESG activities.

Secondly, we also extended the literature on the determinants of corporate ESG practices by providing a new perspective on identifying the motivations behind firms' ESG behaviors by approaching them from an informal external institutional perspective. There has been literature focusing on media coverage's impact on corporate social responsibility and environmental governance. Still, very limited literature focuses on the effects of Internet attention on corporate social and environmental governance behaviors.

Thirdly, by extending the research on the economic impact of online investors' interest from financial markets to corporate governance, the scope and effect of online attention will be enriched, thereby adding new evidence to the "governance effect" of network attention. Furthermore, the paper addresses network attention as a traffic resource that acts as an incentive for firm behavior. The paper also further examines the driving forces behind ESG, demonstrating that online media, like traditional media, form an alternative to formal institutions in performing external governance roles and play an essential role in promoting the development of China's real economy.

2. Literature and Hypothesis development

2.1. The economic consequences of network attention.

Network attention is a hot research topic in behavioral finance. Domestic and international scholars have extensively examined the economic consequences of

network concerns whereby they focused on futures markets (Kou et al. 2018; Han, Li, and Yin 2017), currency markets (Han, Xu, and Yin 2018; Zhang and Wang 2020), commodity markets (Li, Zhang, and Yuan 2019; Yao, Zhang, and Ma 2017) and equity markets (Da, Engelberg, and Gao 2011; Wen et al. 2019). Additionally, certain studies in the literature effectively portrayed that network attention contributed to an essential role in the behavior and decision-making of firms. Concurrently, Han et al. (2018) examined the increase in network attention to reduce inefficient corporate investment. Deng, Feng, and Li (2020) discovered that there was an incentive effect of Internet traffic-based network attention on firm innovation. Feng, Li, and Deng (2021) proposed that the increase in online attention would be able to promote the company's growth significantly. Similarly, a recent study also argued that the increased network attention would significantly enhance corporate green innovation (Li et al. 2022). As such, the attention of investors' networks based on Internet traffic has inevitably emerged as a new resource. It is worth investigating whether it can drive corporate social and environmental governance behavior.

2.2. Factors Influencing ESG

There has been extensive research in the literature on the determinants of corporate ESG performance or CSR from various perspectives, including executive characteristics (Aabo and Giorici 2022; Chen, Zhou, and Zhu 2019; Xu and Ma 2021), operating environment (Chen and Wan 2020; Fu, Ma, and Tian 2021) and intra-firm characteristics factors (Cao, Li, and Xiao 2022; Barros et al. 2022; Drempetic, Klein, and Zwergel 2020). Numerous scholars have also examined the impact of external governance mechanisms on ESG performance. For instance, some studies have shown a strong positive association between media attention to CSR (Zyglidopoulos et al. 2012) and business environmental performance (Su and Fan 2021a). Overall, the above

literature has predominantly focused on traditional media. Likewise, Several scholars have studied the external governance role of online media and demonstrated that online search volume improves corporate environmental performance (Cheng and Liu 2018), carbon information disclosure level (Li et al. 2017), and corporate environmental information disclosure (Fan, Yang, and Liu 2020). Thus, different from information reporting by traditional media organizations, online attention is an active information mining behaviour of investors. As such, it is apparent that the rapid development along with the penetration of the internet in China has been able to offer an excellent opportunity for this paper to further and thoroughly study the external governance role of online media.

2.3. Hypothesis development

Network attention serves as a critical external corporate governance mechanism that monitors and counterbalances corporate management decisions. As the largest group of investors in China's capital market, retail investors are exposed to the threat of profit encroachment from management and major shareholders. Therefore, they would actively gather more information to make accurate investment decisions. The frequency of their keyword searches will reflect the level of interest in the issue (Fan, Yang, and Liu 2020). As soon as a company is brought to the attention of more investors and exposed to the public, current trends in corporate development and investment decisions, personal behavior of executives, and job changes will all be under the scrutiny of the Internet (Feng, Li, and Deng 2021). Investors are not only the recipients of information but also disseminate their attitudes and opinions promptly, rapidly creating pressure on online public opinion (Dyck, Volchkova, and Zingales 2008). Subsequently, this pressure will force companies to adapt their behavior patterns to fit social ethics (Su and Fan 2021b). The principal reason is that ethical business practices will attempt to create

the greatest social return, giving companies legitimacy and easier survival (Nazir et al. 2022). Therefore, companies would encounter barriers to accessing scarce resources if they fail to attain legitimacy. Additionally, online attention can heighten the heat of discussion on key events for listed companies, which may subsequently garner the attention of other external monitoring organizations such as the media, intermediary organizations, and regulators. The intervention of administrative supervision increases the probability of discovering corporate violations which acted as a prior “deterrent” to listed companies. Simultaneously, administrative penalties will heighten the cost of self-interested or opportunistic behavior. Accordingly, managers will cautiously assess the consequences of their non-conforming behavior. when faced with external pressure, the company’s management will greatly increase its willingness to engage in ESG practices in order to gain legitimacy.

Network attention has gradually become an influential traffic resource, which can effectively enhance the exposure rate of listed companies and serve as a publicity role. Investors will not only pay attention to a company’s financial information but also its non-financial performance. ESG performance is one of the key non-financial indicators that investors will focus on when making investment decisions. Internet attention is equivalent to free advertising for a company. Therefore, the company would actively portray more positive images when the network attention is high. Management attaches significant importance to the reputational capital and value of the company in the labor market. On the one hand, reputation is an immensely vital intangible asset of a company that can improve its competitiveness. On the other hand, the market may heavily rely on this capital to infer manager capabilities and determine executive compensation (Ogunfowora, Stackhouse, and Oh 2018). As such, the loss of a company’s reputation will inevitably translate into a lower share price, potentially damaging the company’s

market value (Baker et al. 2021). In a highly competitive industry, the corporate management level usually actively engages in ESG activities and views it as an impression management tool. Simultaneously, certain companies even demonstrate pro-social behavior by falsifying and exaggerating propaganda, actively participating in the battle for Internet traffic by establishing a positive image to cater to the rise in Internet attention. When companies with a high level of online attention encounter negative news, managers commonly tend to engage more in ESG activities to conceal negative company information or minimize damage. Hence, companies attempt to create the illusion of high sustainability by promoting an image of positive contribution to public goods to repair reputational damage. Network attention implies that investors are shareholders or potential shareholders of the company. Therefore, network attention will incentivize companies to engage in ESG practices, attain reputation capital, and achieve optimal publicity.

Network attention can ease corporate financing constraints (Martellini and Menzio 2020). Firstly, investors' profit-seeking mindset and disadvantageous information position would drive them to conduct web searches, pinpoint corporate information, and reduce information asymmetry between investors and companies. Investors' attention is limited, and their external attention is scarce. Therefore, the more they follow a company, the more likely they are to purchase its shares (Zhang, Liao, and Zhang 2014). This potential investment intent enhances the company's ability to raise equity and continue as a going concern. Secondly, network attention increases the willingness of alternative investors and other stakeholders to invest in the business. The high internet "traffic" of online attention will unavoidably generate a wide range of influence and attracts additional stakeholders to the business. With the improvement of corporate social networks, it will be able to assist companies in acquiring more key resources such

as human and financial resources. At the same time, the increased attention of the network screens out adequately-qualified companies and assists them in successfully attaining bank credit opportunities. The tilt of social capital can help in easing financing constraints and enhance the ability of companies to challenge ESG practices. On the contrary, the cost of engaging in ESG practices will increase if companies are at risk of investor divestment or loan disruptions. Eventually, network attention has increased, various types of corporate information have been mined and disclosed, and the transparency of corporate information has significantly increased. Companies actively engage in more ESG activities to demonstrate that they are conscious of their corporate social responsibility and mitigate environmental and governance risks (Liu, Li, and Kong 2022) to boost investor evaluation of the business. A company’s ESG performance will be able to vigorously represent its past performance over time and is more representative of its market outlook, thereby providing investors’ confidence. ESG practices need to be supported by economic strength in addition to the strong will of managers. Concurrently, adequate funding is also necessary for companies to impose ESG changes. As such, network concerns have been alleviating financing constraints, bringing social resource tilt, as well as increasing the companies' willingness and ability to enhance ESG investment.

Therefore, the following hypothesis is proposed. H1: network attention improved corporate ESG practices.

3. Methodology

Considering ESG performance scores’ availability, this study has selected A-share listed companies in China’s Shanghai and Shenzhen markets between 2012 to 2020 as the research subjects. The following principles were followed in the processing of data: (1) The financial sector samples are excluded; (2) The ST or PT categories are excluded; (3)

The samples that possess missing and abnormal data are excluded. Subsequently, a two-sided Winsorize shrinkage at the 1% level for all continuous variables has been successfully performed to effectively prevent the elimination of extreme observations from affecting the empirical results. The final consists of 7,633 firm-year observations corresponding with 7633 listed companies. Simultaneously, the network attention data has been obtained from the CNRDS database, which provides Internet search index values for China's listed companies since 2011. We also successfully acquired the relevant financial data of the company from the China Stock Market and Accounting Research (CSMAR).

3.1. Dependent variables

In consideration of the length of the sample as well as the completeness of the indicator system, the composite ESG score from the Bloomberg database and the individual sub-scores for the fundamental three dimensions of the environment (E), social responsibility (S), and corporate governance (G) have been employed as indicators of ESG practice. This score is a comprehensive assessment of the three dimensions of the enterprise's environment, society, as well as governance based on the corporate social responsibility report, annual report, website and other public information available to investors, and the overall rating of the company is based on the weighted average of the individual scores obtained from the ESG comprehensive score. Correspondingly, the scores will range from 0 to 100, whereby the higher the score, the higher the level of ESG compliance.

3.2. Independent variables

The internet search index effectively measures network attention (ATT), a comprehensive search index calculated based on various internet search data on the

Baidu platform and integrated with relevant information such as news and public opinion. With reference to the previous research literature (Wen et al. 2019), the total value of the search sum for keywords such as stock code, company profile, and the full name of the company has been divided by the number of keywords followed by the natural logarithm to represent the web attention.

3.3. Control variables

Based on the ESG related literature, we choose the following control variables: firm size (Size), firm age (Ln_Age), leverage (Lev), profitability (ROA), management shareholding (MS), the shareholding of institutional investors (INST), share concentration (TOP1), board size (Board), CEO duality (Dual) and corporate property (SOE). All variable definitions are reported in Table A1 in Appendix.

3.4. Empirical model

We utilize the following model to validate the causal relationship between network attention and corporate ESG practices. All regression results have reported robust heteroskedasticity standard errors.

$$ESG_{i,t} = \alpha_0 + \alpha_1 ATT_{i,t} + \alpha_2 Controls_{i,t} + \sum Industry + \sum Year + \varepsilon_{i,t} \quad (1)$$

The independent variable $ATT_{i,t}$ denotes the network attention of firm i in year t. ESG, E, S, and G represent the dependent variable. $ESG_{i,t}$ denotes the total ESG score of firm i in year t or the score of one of the three subscales E, S, and G. $Controls_{i,t}$ represents a set of control variables, ∑Industry denotes the control industry dummy and

∑Year denotes the control time dummy. If the network attention (ATT) coefficient α is positive, then hypothesis 1 holds.

4. Results

4.1. Descriptive statistics

Table 1 depicts the descriptive statistics of all the variables. The mean value of corporate ESG practices is 21.91 and the median is 20.66. Overall, companies' ESG performance has been relatively poor. Among the three sub-index scores, the mean value of corporate governance is 45.05, the mean value of social responsibility is 24.66, and the mean value of environmental performance is 10.88, indicating that corporate governance in the sample has been favorable and there is significant room for improvement in environmental investment. The mean value of network attention (ATT) is 12.09, with a standard deviation of 0.673, indicating that differences exist between investors and attention to different companies.

Table 1. Descriptive statistics of variables

Variables	N	Mean	Std	Min	Max
ESG	7633	21.91	6.248	10.74	49.17
E	7633	10.88	7.682	1.550	49.61
S	7633	24.66	9.048	7.017	57.90
G	7633	45.05	5.086	32.14	58.93
ATT	7633	12.09	0.673	10.40	14.99
Size	7633	23.15	1.292	20.09	26.37
LnAge	7633	2.535	0.587	0	3.332
Lev	7633	0.480	0.199	0.0520	0.905
Roa	7633	0.0420	0.0590	-0.445	0.219
MS	7633	0.0640	0.138	0	0.659

INST	7633	0.535	0.225	0.0100	0.932
Top1	7633	0.372	0.158	0.0810	0.758
Dual	7633	0.185	0.389	0	1
Board	7633	9.045	1.793	5	15
SOE	7633	0.541	0.498	0	1

4.2. Regression analysis

Table 2 reports the results of the impact of network attention on ESG practices. Column (1) reports the estimated network attention coefficient of 0.674, which is significant at the 1% level. In (2), by utilizing the company's reported environmental score E as the dependent variable, the results indicate that an increase in network attention will lead companies to place more emphasis on environmental performance. This has been consistent with the previous findings (Cheng and Liu 2018). In column (3), the regression results in a coefficient of investor attention of 0.396, which is significant at the 10% level. This indicates that increased network attention can motivate companies to engage in more socially beneficial behaviors and are vital participants in the country's development and society. In (4), for corporate governance as the dependent variable, the estimated coefficient of network attention is positively related to G with a coefficient of 0.533, which is significant at the 1% level. This indicates that network attention will effectively motivate the company management to emphasize stakeholders' demands more and strengthen corporate governance in general to maximize shareholders' interests under external supervision. Therefore, it is inevitable that the increase in network attention will lead company managers to be more forward-looking in their strategy formulation and more conducive to the long-term sustainability of the company's growth. Hence, key hypothesis 1 has been fully validated.

Table 2. Principal regression

Variables	ESG	E	S	G
	(1)	(2)	(3)	(4)
ATT	0.674***	0.885***	0.396*	0.533***
	(0.134)	(0.164)	(0.205)	(0.108)
Size	1.661***	1.892***	1.989***	0.738***
	(0.084)	(0.105)	(0.131)	(0.070)
Ln_Age	0.301**	-0.067	-0.348	1.272***
	(0.138)	(0.173)	(0.224)	(0.122)
Lev	-1.850***	-1.419**	-3.486***	-0.668*
	(0.467)	(0.573)	(0.726)	(0.386)
Roa	-1.217	-2.126	1.071	-2.451**
	(1.189)	(1.477)	(1.774)	(1.048)
MS	5.678***	7.210***	6.758***	0.311
	(0.693)	(0.830)	(1.118)	(0.598)
INST	5.699***	6.506***	5.136***	4.270***
	(0.515)	(0.641)	(0.815)	(0.437)
Top1	-2.428***	-3.232***	-2.625***	-0.514
	(0.556)	(0.676)	(0.879)	(0.465)
Dual	-0.343**	-0.110	-0.870***	-0.024
	(0.165)	(0.202)	(0.253)	(0.144)
Board	-0.022	-0.039	0.059	-0.052
	(0.039)	(0.047)	(0.062)	(0.033)
SOE	0.699***	0.708***	1.105***	0.231
	(0.167)	(0.210)	(0.245)	(0.144)
Constant	-26.758***	-46.880***	-24.167***	18.107***
	(1.944)	(2.447)	(2.855)	(1.585)
Year/ Industry F.E.	Yes	Yes	Yes	Yes
Observations	7,633	7,633	7,633	7,633
R-squared	0.266	0.256	0.159	0.233

Notes: ***, **, and * mean significance level at the 1, 5 and 10%, respectively. Values in parentheses are heteroskedasticity robust standard errors.

4.3. Robustness test

4.3.1. Instrumental variable regression.

We refer to (Yang, Chen, and Liu 2017) for selecting instrumental variables and use the proportion of shares outstanding (Trade) as the instrumental variable of network attention. Table 3 column (1) displays the results of the first stage regression, where shares outstanding (Trade) and network attention are positively correlated, further demonstrating the relevance of the instrumental variables. Column (2) indicate that network concerns remain significantly and positively associated with ESG practices after controlling for endogeneity issues. Also, we conducted a weak instrumental variable test, and the F-statistic value was 299.609, which is greater than the critical value of 16.380 under the 10% criterion, meaning that the selected instrumental variables are relatively reliable.

4.3.2. Replace key variables

We use the summed logarithm of search values for keywords such as stock code, company profile, and full company name from the Web search index database as an alternative measure of network attention (IA). Table 3 column (3) shows that the regression coefficients of the explanatory variables are significant at the 1% level, and the findings remain robust.

4.3.3. Change lag time

Firms need time to adjust their investment policies dynamically, so this study uses lagged one-period network attention and related control variables, which can also

mitigate potential endogenous problems. Table 3 column (4) shows a significant positive relationship between network attention and ESG practices.

4.3.4. Control individual fixed effects

To mitigate the potential problem of omitted variables that may arise from ignoring firm-specific characteristics, Eq. (1) is re-estimated using a model that includes firm-fixed and year-fixed effects. Table 3 column (5) shows that network attention is significantly and positively related to ESG practices at the 1% level, indicating that higher investor network attention increases higher ESG inputs by firms, and the study findings remain unchanged.

Table 3. Robustness test

Variables	ATT	ESG	ESG	ESG	ESG
	(1)	(2)	(3)	(4)	(5)
ATT		9.970***			0.585***
		(2.086)			(0.141)
Trade	0.218***				
	(0.034)				
IA			0.530***		
			(0.140)		
ATT1				0.719***	
				(0.151)	
Other Controls	Yes	Yes	Yes	Yes	Yes
Constant	6.216***	-83.550***	-25.731***	-28.933***	-34.511***
	(0.163)	(13.678)	(1.943)	(2.138)	(12.320)
Year F.E.	Yes	Yes	Yes	Yes	Yes
Industry F.E.	Yes	Yes	Yes	Yes	No
Code F.E.	No	No	No	No	Yes
Observations	7,010	7,010	7,633	6,480	7,010

Notes: ***, **, and * mean significance level at the 1, 5 and 10%, respectively. Values in parentheses are heteroskedasticity robust standard errors. Due to space constraints, the results of the robustness tests for the three ESG sub-dimensions are not reported but are kept for reference.

5. Further analysis

5.1. The heterogeneity analysis of the marketization process

China's comprehensive national power continues to develop and is progressively transitioning from a planned economy to a market economy, which is a significant institutional background influencing enterprise micro-behavior. On the one hand, higher marketization implies less government intervention and a high level of investor protection. Therefore, retail investors will actively participate in the capital market, seek information via the network, and increase the company network's attention. As such, companies will implement more ESG initiatives to reduce financing costs and demonstrate the value of long-term corporate growth. On the other hand, regions with a higher marketization process typically possess a more developed legal environment and greater information transparency. Hence, investors will have higher expectations for corporate ESG and can effectively monitor and regulate corporate behavior through online concerns. We employ the marketization index developed by Wang et al. (2019) to determine each location's marketization level effectively. In Eq. (1), we introduce an interaction term (ATT×Market) to investigate the moderating effect of the marketization process comprehensively.

The results in column (1) of Table 4 demonstrate that the coefficient of the cross-product term is 0.176 and is significant at the 1% level. This implies that the higher the marketization process, the larger the positive influence of network attention on ESG practices.

Table 4. The moderating effect of the marketization process

Variables	ESG	E	S	G
	(1)	(2)	(3)	(4)
ATT	0.724***	0.928***	0.475**	0.573***
	(0.133)	(0.164)	(0.203)	(0.109)
ATT×Market	0.176***	0.160**	0.258***	0.135***
	(0.056)	(0.070)	(0.087)	(0.046)
Market	0.378***	0.448***	0.535***	0.140***
	(0.041)	(0.050)	(0.068)	(0.034)
Other Controls	Yes	Yes	Yes	Yes
Constant	-29.323***	-49.548***	-28.291***	16.893***
	(1.992)	(2.509)	(2.909)	(1.626)
Year/ Industry F.E.	Yes	Yes	Yes	Yes
Observations	7,585	7,585	7,585	7,585
R-squared	0.275	0.264	0.168	0.236

Notes: ***, **, and * mean significance level at the 1, 5 and 10%, respectively. Values in parentheses are heteroskedasticity robust standard errors.

5.2. Heterogeneity of industry competition

First, in the low-competition industry, the market concentration is high and some companies are in a monopoly position. Companies face less competitive pressure and have more stable capital and resources. When the network attention is raised, it has more strength to make ESG investments. Second, when an industry is concentrated in a few large enterprises, other businesses have fewer opportunities to gain market share, while monopolies have more opportunities to gain network attention. Companies will take advantage of the opportunity to increase web traffic and investor recognition by engaging in ESG activities for corporate promotion. Finally, in low-competition industries, where the degree of information misalignment is greater, network attention, an external governance mechanism, may play a greater role and better stimulate corporate ESG

practices. We use the HHI, the cumulative square of the ratio of each firm's primary business revenue to the industry's total primary business revenue, to assess the level of competition to which a firm is subject. A higher value indicates that there is less industry competition. Table 5 demonstrates that ATT×HHI coefficients are all significantly positive, indicating that the effect of network attention on improving ESG practices is more pronounced in industries with little competition.

Table 5. Regulating the role of industry competition

Variables	ESG	E	S	G
	(1)	(2)	(3)	(4)
ATT	0.653***	0.859***	0.377*	0.524***
	(0.134)	(0.164)	(0.206)	(0.108)
ATT×HHI	3.482***	3.961***	4.282***	1.325**
	(0.726)	(0.894)	(1.183)	(0.557)
HHI	-1.605	-1.172	-3.696**	-0.456
	(1.087)	(1.278)	(1.855)	(1.013)
Other Controls	Yes	Yes	Yes	Yes
Constant	-26.214***	-46.383***	-23.174***	18.287***
	(1.954)	(2.462)	(2.881)	(1.601)
Year/ Industry F.E.	Yes	Yes	Yes	Yes
Observations	7,632	7,632	7,632	7,632
R-squared	0.268	0.258	0.161	0.233

Notes: ***, **, and * mean significance level at the 1, 5 and 10%, respectively. Values in parentheses are heteroskedasticity robust standard errors.

5.3. Heterogeneity of property rights

In the Chinese institutional environment, where the nature of property rights is an essential characteristic of firms, we further examine whether differences existed in network attention about corporate ESG practices among SOEs and non-SOEs. On the one

hand, as SOEs possess a strong political connection with the government and bear the responsibility of maintaining social stability as well as enhancing people's well-being, thus they are required to set an exemplary example of ESG practices at the national level. Hence, the ESG performance of SOEs is often political and mandatory, and regardless if they receive more attention from investors, the ESG investment of SOEs is comparatively positive. Non-SOEs, whose main business goal is to improve economic efficiency, are hardly willing to take the initiative to undertake ESG practices. After all, this investment will not bring objective benefits to the enterprise in the short term. However, when network attention is raised, non-state companies are more proactive in engaging in ESG to gain legitimacy and cater to government policy guidance. Hence, we can infer that ESG practices are more pronounced among non-SOEs when network attention are raised. The ESG×SOE cross-product term is added to Eq. (1) and then regressed. Table 6 presents that the regression coefficients of the cross-product terms are significantly negative, except for column (3), which is not significant, which is consistent with the inference that property rights possess a negative moderating effect.

Table 6. Regulating the role of property rights

Variables	ESG	E	S	G
	(1)	(2)	(3)	(4)
ATT	0.859***	1.226***	0.284	0.728***
	(0.176)	(0.217)	(0.257)	(0.144)
ATT×SOE	-0.333*	-0.614**	0.203	-0.352**
	(0.199)	(0.247)	(0.309)	(0.166)
SOE	4.729**	8.142***	-1.354	4.493**
	(2.400)	(2.970)	(3.748)	(2.009)
Other Controls	Yes	Yes	Yes	Yes
Constant	-29.123***	-51.241***	-22.725***	15.607***

	(2.434)	(3.114)	(3.449)	(1.999)
Year/ Industry F.E.	Yes	Yes	Yes	Yes
Observations	7,633	7,633	7,633	7,633
R-squared	0.266	0.257	0.159	0.233

Notes: ***, **, and * mean significance level at the 1, 5 and 10%, respectively. Values in parentheses are heteroskedasticity robust standard errors.

6. Discussion and conclusions

Network attention has played an important informal external governance role in affecting corporate investment behavior. Based on 7633 firm-level observations in China from 2012 to 2020, we attempt to assess the potential impact of network attention on ESG. The following significant conclusions are drawn: First, network attention improves corporate ESG practice significantly. Furthermore, network attention is significantly and positively related to all three major ESG sub-dimensions, namely, E, S, and G. Second, the network attention on promoting corporate willingness and ability to practice ESG through external monitoring, image enhancement incentives, and alleviation of financing constraints. Finally, in a higher marketization process, severely competitive industries and non-state enterprises, the influence of network attention on corporate ESG practices has been greater.

This paper extends the body of literature about the impact of external governance on corporate investment behaviors. ESG practices align with current national development policies and are vital to consumers, investors, as well as other stakeholders. Some scholars equate ESG with social responsibility (Sabbaghi 2020), but we believe ESG differs from CSR, especially its connotations.

Most previous research focused on the relationship between ESG and financial performance and market value indicators (Bruna et al. 2022; Velte 2017). Understanding the motivations behind ESG practices is critical for improving long-term corporate

sustainability. We investigated the impact of network attention on corporate ESG practices filling the gaps from an investor's perspective. Scholars are studying the connection between web search volume and corporate environmental performance (Cheng and Liu 2018), as well as environmental information disclosure (Fan, Yang, and Liu 2020), but only one of the environmental dimensions of ESG practices is taken into consideration. Also, their findings are supported by our results, which manifest that network attention is significantly positive for E. Simultaneously, this paper investigates ESG both from a comprehensive and a segmented perspective, which is consistent with China's current strategic requirement of transitioning from uneven to balanced development, as well as achieving coordination, unity, and sustainable development goals in all aspects of corporate practice.

The media is an important external monitoring mechanism that can significantly improve CSR (Zyglidopoulos et al. 2012). Unlike traditional media, where investors passively receive information, we believe online investor attention plays an important external monitoring role and significantly improves corporate ESG practices. Previous literature has paid attention to stakeholder theory, institutional perspective, etc., arguing that corporate CSR is about gaining and retaining legitimacy. Investors can actively cultivate and search for corporate information thanks to the rapid development of digital networks, and online attention has a significant traffic effect. On this basis, we also considered that the traffic generated by network attention motivates people to participate in ESG for image promotion. Furthermore, the network attention relieved corporate financing constraints and provided adequate financial support for corporate ESG practices.

This study has important managerial implications. For enterprises, we cannot merely pursue the goal of "profit maximization" for businesses while ignoring long-term

development. Companies should actively engage in ESG practices as a "value investment" that earns them a reputation and their stakeholders' trust and support. At the government level, we should actively increase network platform construction and strengthen network ecology guidance and governance.

Appendix A

Table A1. Variable definitions

Variable	Variable Definition
ESG	Total Corporate ESG Performance Score.
E	Environmental score for ESG performance.
S	Social Responsibility Score for ESG Performance.
G	Corporate Governance Score for ESG Performance.
ATT	Natural logarithm of the (index value /keyword number).
Size	Natural logarithm of the (total asset)
Ln Age	Natural logarithm of the year of listing.
Lev	Total liabilities divided by total assets.
ROA	Net profit divided by total assets.
M.S.	Management shareholding data divided by total share capital
INST	The percentage of shares hold by the institutional investors.
Top1	The total shareholding ratio of the top ten shareholders.
Dual	Equals1if CEO is also the chairman of the board, and 0 otherwise.
Board	Number of Directors.
SOE	Equals to 1 for state-owned enterprises, 0 otherwise.
Industry	According to the "Industry Classification Guidelines" by China Securities Regulatory Commission, we divide the listed companies by sector.
Year dummy	Set reference variables based on the years 2012-2020

References

Aabo, T., and I. C. Giorici. 2022. "Do female CEOs matter for ESG scores?". *Global Finance Journal*:100722. doi: 10.1016/j.gfj.2022.100722.

Baker, A. N., D. King, M. Nalick, M. Tempio, V. K. Gupta, and C. A. Pierce. 2021. "Managers' sexually-oriented behavior and firm performance: linking media reports to stock market reactions and legal risk." *Journal of Strategy and Management*.

Barber, B. M., and T. Odean. 2008. "All that glitters: The effect of attention and news on the buying behavior of individual and institutional investors." *The Review of Financial Studies* 21 (2):785-818.

Barros, V., P. V. Matos, J. M. Sarmento, and P. R. Vieira. 2022. "M&A activity as a driver for better ESG performance." *Technological Forecasting and Social Change* 175:121338.

Bruna, M. G., S. Loprevite, D. Raucci, B. Ricca, and D. Rupo. 2022. "Investigating the marginal impact of ESG results on corporate financial performance." *Finance Research Letters* 47:102828.

Cao, J., W. Li, and S. Xiao. 2022. "Does mixed ownership reform affect private firms' ESG practices? Evidence from a quasi- natural experiment in China." *Financial Markets, Institutions & Instruments* 31 (2-3):47-86.

Chen, W. T., G. S. Zhou, and X. K. Zhu. 2019. "CEO tenure and corporate social responsibility performance." *Journal of Business Research* 95:292-302.

Chen, X. y., and P. Wan. 2020. "Social trust and corporate social responsibility: Evidence from China." *Corporate Social Responsibility and Environmental Management* 27 (2):485-500.

Cheng, J., and Y. Liu. 2018. "The effects of public attention on the environmental performance of high-polluting firms: Based on big data from web search in China." *Journal of Cleaner Production* 186:335-341.

Da, Z., J. Engelberg, and p. j. Gao. 2011. "In search of attention." *The journal of finance* 66 (5):1461-1499.

Deng, X. r., X. l. Feng, and Z. w. Li. 2020. "The Research on Influencing Mechanism of Network Attention on the Incentive Effect of Corporate Innovation: An Empirical Analysis Based on the Data of China' s A-share Listed Companies." *Journal of Central University of Finance & Economics* (9):93-106. doi: 10.19681/j.cnki.jcufe.2020.09.010.

Drempetic, S., C. Klein, and B. Zwergel. 2020. "The influence of firm size on the ESG score: Corporate sustainability ratings under review." *Journal of Business Ethics* 167 (2):333-360.

Dyck, A., N. Volchkova, and L. Zingales. 2008. "The corporate governance role of the media: Evidence from Russia." *The journal of finance* 63 (3):1093-1135.

Fan, L., K. l. Yang, and L. p. Liu. 2020. "New media environment, environmental information disclosure and firm valuation: Evidence from high-polluting enterprises in China." *Journal of Cleaner Production* 277:123253.

Feng, X., Z. Li, and X. Deng. 2021. "Network Attention and Enterprise Growth- Based on the Perspective of Enterprise Life Cycle ". *Modern Economic Science* 43

(5):128-140.

Fu, P., C. Ma, and Y. Tian. 2021. "Confucianism and corporate ESG scores: evidence from China." *Available at SSRN 3963151*.

Han, L., Z. Li, and L. Yin. 2017. "The effects of investor attention on commodity futures markets." *Journal of Futures Markets* 37 (10):1031-1049.

Han, L., Y. Xu, and L. Yin. 2018. "Does investor attention matter? The attention-return relationships in FX markets." *Economic Modelling* 68:644-660.

Han, S., Y. Pan, H. Li, and l. Li. 2018. "Network Media Attention，External Environment and Inefficient Investment: Based on the Information Effect and Supervision Effect." *China Economic Studies* (1):73-85. doi: 10.19365/j.issn1000-4181.2018.01.07.

Kou, Y., Q. Ye, F. Zhao, and X. Wang. 2018. "Effects of investor attention on commodity futures markets." *Finance Research Letters* 25:190-195.

Li, L., Q. Liu, D. Tang, and J. Xiong. 2017. "Media reporting, carbon information disclosure, and the cost of equity financing: evidence from China." *Environmental Science and Pollution Research* 24 (10):9447-9459.

Li, M., N. Li, M. A. Khan, N. Khaliq, and F. U. Rehman. 2022. "Can retail investors induce corporate green innovation?-Evidence from Baidu Search Index." *Heliyon*:e09663.

Li, S., H. Zhang, and D. Yuan. 2019. "Investor attention and crude oil prices: Evidence from nonlinear Granger causality tests." *Energy Economics* 84:104494.

Liu, x. x., h. y. Li, and x. x. Kong. 2022. "Research on the Influence of Party Organization Governance on Enterprise ESG Performance." *Collected Essays on Finance and Economics* (1):100-112. doi: 10.13762/j.cnki.cjlc.2022.01.008.

Martellini, P., and G. Menzio. 2020. "Declining search frictions, unemployment, and growth." *Journal of Political Economy* 128 (12):4387-4437.

Nazir, M., M. Akbar, A. Akbar, P. Poulovo, A. Hussain, and M. A. Qureshi. 2022. "The nexus between corporate environment, social, and governance performance and cost of capital: evidence from top global tech leaders." *Environmental Science and Pollution Research* 29 (15):22623-22636.

Ogunfowora, B., M. Stackhouse, and W. Y. Oh. 2018. "Media depictions of CEO ethics and stakeholder support of CSR initiatives: The mediating roles of CSR motive attributions and cynicism." *Journal of Business Ethics* 150 (2):525-540.

Sabbaghi, O. 2020. "The impact of news on the volatility of ESG firms." *Global Finance Journal*:100570.

Su, W., and Y.-H. Fan. 2021a. "Impact of media attention on environmental performance in China." *Environmental Challenges* 4:100133.

Su, W., and Y. H. Fan. 2021b. "Impact of media attention on environmental performance in China." *Environmental Challenges* 4:100133.

Velte, P. 2017. "Does ESG performance have an impact on financial performance? Evidence from Germany." *Journal of Global Responsibility* 8 (2):169-178.

Wen, f. h., l. h. Xu, g. d. Ouyang, and g. Kou. 2019. "Retail investor attention and stock price crash risk: evidence from China." *International Review of Financial Analysis* 65:101376.

Xu, S., and P. Ma. 2021. "CEOs' Poverty Experience and Corporate Social Responsibility: Are CEOs Who Have Experienced Poverty More Generous?". *Journal of Business Ethics*:1-30.

Yang, d. g., h. w. Chen, and q. l. Liu. 2017. "Media Pressure and Corporate Innovation." *Economic Research Journal* 52 (8):125-139.

Yao, T., Y. J. Zhang, and C. Q. Ma. 2017. "How does investor attention affect international crude oil prices?". *Applied Energy* 205:336-344.

Zhang, J. d., w. Liao, and r. w. Zhang. 2014. "The Effect of Ordinary Investors' Attention on Volume and Price of Stock Market: Empirical Evidence based on Baidu Index." *Accounting Research* (8):52-59, 97.

Zhang, W., and P. f. Wang. 2020. "Investor attention and the pricing of cryptocurrency market." *Evolutionary and Institutional Economics Review* 17 (2):445-468.

Zyglidopoulos, S. C., A. P. Georgiadis, C. E. Carroll, and D. S. Siegel. 2012. "Does media attention drive corporate social responsibility?". *Journal of Business Research* 65 (11):1622-1627.
